# SARS-CoV-2 RT-PCR to Screen for B.1.617.2 (Delta) Variant of Concern

**DOI:** 10.3390/diagnostics12092056

**Published:** 2022-08-24

**Authors:** Kym Lowry, Claire Wang, Amanda Bordin, Cameron Buckley, Steven Badman, Patrick Harris, Ian Mackay, David Whiley

**Affiliations:** 1 The Queensland Paediatric Infectious Diseases (QPID) Research Group, Queensland Children’s Hospital, Brisbane, QLD 4101, Australia; 2 The University of Queensland Centre for Clinical Research (UQCCR), Faculty of Medicine, The University of Queensland, Brisbane, QLD 4029, Australia; 3 Infectious Diseases Laboratory, Prevention Division, Pathology Queensland, Brisbane, QLD 4006, Australia; 4 Kirby Institute for Infection and Immunity in Society, University of New South Wales (UNSW) Medicine, UNSW Sydney, Kensington, Sydney, NSW 2052, Australia; 5 Prevention Division, Pathology Queensland, Brisbane, QLD 4006, Australia; 6 Child Health Research Centre, The University of Queensland, Brisbane, QLD 4029, Australia

**Keywords:** SARS-CoV-2, RT-PCR, variant assay, variant of concern, Delta, whole genome sequencing

## Abstract

The continuous transmission and evolution of severe acute respiratory syndrome coronavirus 2 (SARS-CoV-2) has required that diagnostic capabilities be constantly monitored and updated as new variants emerge and prior variants disappear. Although whole genome sequencing provides full characterisation of SARS-CoV-2 directly from patient samples, this has limited throughput and requires sufficient resources. To enhance screening for circulating variants, we designed a rapid in-house RT-PCR assay to target a spike mutation (D950N) in Delta variants, which is not detected in the remaining variants of concern (VOCs). Assay sensitivity for detecting Delta variants was 93% and specificity was 100% using a sequenced sample bank of several lineages. As the D950N mutation is prevalent in >95% of the global Delta variant sequences deposited in GISAID, this assay has the potential to provide rapid results to determine if the samples are presumptively Delta variants and can support clinicians in timely clinical decision-making for effective treatments and surveillance.

## 1. Introduction

Severe Acute Respiratory Syndrome Coronavirus 2 (SARS-CoV-2) variants have continually emerged due to ongoing transmission and evolution of this virus globally. Since the pandemic was first declared in March 2020 by World Health Organisation (WHO) [1], there have been surge outbreaks attributed to Variants of Concern (VOC) as classified by the WHO; including B.1.17 (Alpha), B.1.351 (Beta), P.1 (Gamma), B.1.617.2 (Delta), and B.1.1.529 (Omicron) [2,3,4,5,6,7]. The WHO has outlined three working definitions of a VOC, comprising; increased transmissibility or detrimental change in COVID-19 epidemiology; OR an increase in virulence or change in clinical disease presentation; OR a demonstrated decrease in effectiveness of public health and social measures or available diagnostics, vaccines, or therapeutics [6]. Whole genome sequencing (WGS) of SARS-CoV-2 in patient samples has proved crucial for genetic surveillance by detecting and monitoring changes in circulating strains and remains the gold standard for VOC detection. Notably, the rapid sharing of SARS-CoV-2 genomic sequences and related clinical and epidemiological data to The GISAID Initiative has made it possible to track current strains in real-time [8], and other online databases, such as *outbreak.info*, *nextstrain.org, and cov-lineages.org*, have combined genetic SARS-CoV-2 data from several data sources (including The GISAID Initiative) to provide essential analysis of sequence variability and geo-temporal analysis [9,10].

While WGS is a powerful resource for detecting and monitoring circulation patterns of variants, it is typically restricted to sophisticated reference laboratories with the required facilities, expertise and workflows in place and is therefore not ideal in smaller, resource-limited regional laboratories. To supplement WGS detection of VOC, various PCR-based methods for targeted rapid detection of VOCs have been described [11,12,13], and in fact there are now several commercial SARS-CoV-2 VOC kits available, primarily as Research Use Only (RUO) tests (Table 1). Such commercial and in-house VOC PCR assays can compare and define persistent versus re-infections in patients and define a lineage in low load samples that may not successfully sequence using WGS platforms. Due to their simplicity and rapid turnaround time, VOC PCR assays can be advantageous for specific detection of certain VOCs, particularly where a rapid result is clinically useful for treatment considerations. Rapid VOC PCR assays can play a part where the variable efficacy of monoclonal antibodies or antiviral treatments can impact patient outcomes depending on the infecting SARS-CoV-2 lineage. At the time of conducting this study, the initial Omicron wave was reaching a peak in Australia (and elsewhere) but Delta lineages were still circulating. At least three clinical monoclonal antibodies (mAbs) are effective treatments for Delta infection but less effective for Omicron variants; Etesevimab, Casirivimab, and Imdevimab [14]. To enable rapid screening for a presumptive Delta infection, we developed and validated an RT-PCR based method for direct detection of Delta lineages in clinical samples. In doing so, we sought to ensure optimal specificity for Delta variant by targeting D950N (G24410A) mutation in the Spike coding region of SARS-CoV-2.

## 2. Materials and Methods

### 2.1. RT-PCR for Detection of SARS-CoV-2 Delta VOC (D950N-PCR)

At the time of writing, the D950N (G24410A) mutation was detected in over 95% of Delta sequences submitted in GISAID (4,199,101/4,432,059), and <0.1% of remaining VOC sequences collectively (7964/11,055,194) [9]. Detection of the D950N spike mutation (G24410A) was performed using allelic discrimination with a single set of primers to amplify the target region combined with two sequence specific probes for differentiating D950 (other strains/variants) and D950N mutation (Delta variants). A set of primers and probes were designed based on the Wuhan-Hu-1 sequence (GenBank accession MN908947) and available sequences deposited in GISAID. Briefly, the D950N-PCR RT-PCR reaction mix consisted of QIAGEN OneStep RT-PCR Kit (Qiagen, Australia); 0.4 µM of forward (GTGCAGGTGCTGCATTACAAA) and reverse (AGCCTCAACTTTGTCAAGACG) primers, 0.2 µM of locked nucleic acid (LNA; LNA bases depicted in the sequence using a preceding ‘+’) probes D950-WT (6FAM-ACTT+CA+A+G+ATGTGG-BHQ1) and D950N-mut (HEX-CTT+CAA+A+AT+GT+GGTC-BHQ1), and 2.5 µL of nucleic acid extract, made up to a final reaction volume of 25 µL using DNase-free water. Amplification and detection were performed on the Rotor-Gene Q (Qiagen, Australia), with an initial reverse transcription step of 50 °C for 30 min, a 95 °C activation for 15 min followed by 45 cycles at 94 °C for 15 s and 60 °C for 1 min each cycle. Due to labelling of the LNA probes, reactions could be distinguished using the Rotor-Gene Q (Qiagen, Australia) green and yellow channels, for D950 and D950N mutation, respectively.

A probe designed to detect strains/variants other than Delta sequence (no known mutations at the D950 site) was included in the D950N Delta assay to ensure that a negative mutation result was due to the presence of D950 and not a result of low SARS-CoV-2 load in the patient sample extract. Based on current sequence data, the spike D950N mutation is conserved to Delta and Mu lineages and is located in a conserved region outside the receptor-binding domain (RBD) which is not prone to high mutation rates [15]. At the time of writing, D950N spike mutation is present in 4,199,101/4,432,059 (94.7%) of Delta sequences, 13,732/15,537 (88.4%) of Mu sequences, and only present in 7964/11,055,194 (0.07%) of the remaining sequences previously submitted to GISAID [8].

### 2.2. Patient Specimens

The assay was validated using a total of 286 specimens submitted to the Infectious Diseases Laboratory, Pathology Queensland for SARS-CoV-2 screening. All samples were nasopharyngeal/oropharyngeal swabs collected between March 2020 to March 2022 and represented mostly symptomatic patients of varying age groups from the general population who presented for testing to local Queensland COVID-19 fever clinics. Patient nasopharyngeal/oropharyngeal dry swabs were resuspended in 1.5 mL of PBS and nucleic acids were extracted using an MGISP-960 instrument and MGIEasy Nucleic Acid extraction kit (MGI, Australia) prior to SARS-CoV-2 amplification using the validated Real-Time Fluorescent RT-PCR Kit for Detecting SARS-CoV-2 (BGI, Australia) at the Infectious Diseases Laboratory, Pathology Queensland. Remnant nucleic acid extract from SARS-CoV-2 screening was then used for the D950N-PCR, as described above.

Genomic data was available for 75 patient specimens, positive for SARS-CoV-2, and confirmed by WGS performed at Forensic and Scientific Services, Queensland Health or University of Queensland Centre for Clinical Research (UQCCR), Brisbane, Australia (Table 2). Whole genome sequencing at UQCCR was conducted using Illumina^®^ COVIDSeq™ RUO with DRAGEN App analysis.

These samples have been collected throughout the pandemic, spanning from March 2021 to April 2022, and were selected to test assay specificity; these included non-VOC strains; B.1.1 (n = 1), B.1 (n = 1), B.6.8 (n = 1), B.1.466.2 (n = 3), and VOC strains; Alpha (n = 6), Beta (n = 3), Delta (n = 28), and Omicron (n = 32) lineages.

The remaining 211 sample cohort consisted of SARS-CoV-2 positive (n = 150) and SARS-CoV-2 negative (n = 61) patient specimens as previously tested by Pathology Queensland, were all untyped, and were included in the study to provide an overall characterisation rate for the D950N-PCR. These samples were not included in specificity evaluations but were included to show that the assay could correctly call positive or negative SARS-CoV-2 samples with either D950/D950N or no detection. This cohort was selected from two specific time periods to capture maximum positive patient samples (January 2022), and a mixture of positive and negative patient samples (February 2022).

### 2.3. Performance of the VOC Assay

The performance of the assay was assessed for sensitivity and specificity. The limit of detection (LoD) of the D950N-PCR was determined by testing 10-fold dilutions of cultured SARS-CoV-2 clinical viral isolate extract RNA with a known titration of TCID50/mL in duplicate [16]. As this assay is intended for use as a reflex test following a positive SARS-CoV-2 detection, evaluation of specificity to other respiratory viruses such as rhinoviruses and influenzavirus was not performed. Clinical specificity was determined using the sequenced sample bank of patient specimens [n = 75], with the presence or absence of D950N in the genetic sequences providing a measure of assay concordance.

## 3. Results

Of the 75 WGS-confirmed patient sample extracts tested with the D950N-PCR Delta assay, 96% (72/75) did not have the mutation (D950) [n = 46] while [n = 26] had the mutation (D950N) [n = 26] (Table 2). Uncharacterised samples by the D950N-PCR were classified as non-detection for both non-mutant and mutant probe [n = 3] and showed low viral loads for detection in RT-PCR. From the characterised samples there was 100% concordance between the expected RT-PCR result and sequence results by WGS, as only VOC-Delta samples contained the D950N mutation. For these samples, the original SARS-CoV-2 detection cycle threshold (Ct) values using the BGI Real Time Fluorescent RT-PCR Kit ranged from 13.75–30.51 and 13.64–30.13 cycles, median 15.32 and 14.94 cycles for N and ORF1ab targets, respectively. Uncharacterised samples by the D950N-PCR were classified as non-detection for both non-mutant and mutant probe. All known SARS-CoV-2 positive samples (1 pre-VOC, 1 Beta, 2 Delta, and 4 presumptive Omicron) that were not detected in the assay had Ct values of 30.81 and 30.57 cycles or higher for N and ORF1ab targets, respectively in the BGI assay which was consistent with lower viral loads (Supplementary Table S1). The D950N-PCR assay detected 26/28 (93%) Delta strains in the study via the D950N probe, while two remained uncharacterised due to low viral load and all other 46 characterised samples (all of which were non-Delta strains) provided signal in the non-mutant D950 probe only. Therefore, the clinical sensitivity of D950N-PCR in detecting Delta in the sample bank was 93% (95% CI 76.27–99.1) and of those, 100% (95% CI 96.37–100) were specific to Delta or non-Delta detections as confirmed by sequencing

For the LoD of the D950N-PCR, we used serially diluted (10-fold) extract derived from SARS-CoV-2 culture originally at 0.5 × 10^7^ TCID50/mL, with dilutions assayed in duplicate. Using the Rotor-Gene Q (Qiagen, Australia), the LoD of replicates was 2 × 10^3^ TCID50/mL with the highest detectable Ct value of 37.625 for D950 probe.

To determine a broader application of the D950N-PCR, we tested a further 211 patient specimens (both positive and negative SARS-CoV-2 status known but VOC status unknown) to provide a characterisation rate for this sample bank (n = 211). Overall, the assay detected 94.67% (95% CI 89.67–97.43) of positive clinical samples which were called as either D950 or D950N.

Limitations of this study: Due to limited availability of positive SARS-CoV-2 patient specimens during the early stages of the pandemic, some nucleic acid extracts were diluted to ensure sufficient material for validation, therefore affecting sensitivity. Sequencing data was not available for all samples, limiting the ability to fully explore assay specificity using a gold standard. Likewise, not all variants have been tested in this study, and it is important to note that D950N has been reported in other variants. For example, almost 88% of Mu sequences in GISAID contain a D950N mutation 13,732/15,537 (88.4%) but this lineage has not been detected since early October 2021 [WHO: Tracking SARS-CoV-2 variants website. accessed on 9 April 2022]. D950N has also been reported, albeit rarely, among Gamma variants; of 121,559 genome sequences deposited to GISAID, the D950N mutation is only detected in 14 of those sequences (0.01%). Finally, it should be noted that the utility of these types of VOC PCRs can evolve with changes in circulating strains. For instance, circulation of Delta VOC has almost disappeared in Australia since the assay was first developed. New VOC PCR assays will require constant updating and re-evaluation for specificity as new variants emerge and prior variants disappear from circulation, however if limitations of assays are monitored and understood and supported by full WGS characterisation where needed, then they still have a role to play in providing broad epidemiological data. Such VOC PCR assays provide mechanisms for comparing and defining persistent infection versus re-infection in a patient and enables a cheap screening mechanism for laboratories that are unable to support NGS facilities.

## 4. Discussion

RT-PCR assays specifically to detect VOCs can provide rapid and clinically useful data for treatment considerations, particularly if variable efficacy is demonstrated for mAb therapies and antivirals. For instance, in vitro studies have demonstrated the loss of activity for bamlanivimab against the Delta variant, yet combinations of mAbs (i.e., estesevimab with bamlanivimab, imdevimab with casirivimab, and tixagevimab with cilgavimab) are able to neutralise Delta variants to provide treatment options [14,17]. Alternatively, the combinatorial use of antivirals, molnupiravir and nirmatrelvir, is supported by in vitro models despite subtle differences in antiviral responses among the original Wuhan strain, Delta, and Omicron lineages [18].

Although commercial RT-PCR assays also provide high clinical and analytical performances at/or approaching 100% for sensitivity and specificity, the implementation of such assays is impacted by slower industry development and validation, as well as larger scale manufacturing and global distribution [19,20]. Rapid and local laboratory developed assays have been designed and validated to detect circulating SARS-CoV-2 variants quickly with high overall sensitivities approaching 100% and 100% specificity for all [21,22]. Laboratories can design and develop updated RT-PCR assays as new variants emerge without the need to wait for similar commercial assay amendments.

Here, we developed and validated an RT-PCR assay capable of distinguishing the presence and absence of D950N mutation in SARS-CoV-2 positive samples. The assay sensitivity and specificity were determined to be 93% and 100%, respectively, using a sequenced sample bank. The D950N mutation is prevalent in >95% of the Delta variant samples and is a specific mutation marker for Delta VOC. The assay has the potential to provide rapid results to determine if the sample isa presumptive Delta variant, and to inform clinicians to make timely treatment decision quickly.

## Figures and Tables

**Table 1:** Commercial SARS-CoV-2 variant kits (RUO) and mutations detected.

Kit	VOC	Mutations Detected
cobas^®^ SARS-CoV-2 Variant Set 1 (RUO) (Roche)	Alpha, Beta, Gamma, Iota, Mu, Omicron	N501Y, E484K, del69-70 *
PlexPrime^®^ SARS-CoV-2 L452Q (SpeeDx Pty Ltd., Eveleigh, NSW, Australia)	Lambda	L452Q
PlexPrime^®^ SARS-CoV-2 P681R (SpeeDx Pty Ltd.)	Delta	P681R
PlexPrime^®^ SARS-CoV-2 Alpha/Beta/Gamma + (SpeeDx Pty Ltd.)	Alpha, Beta, Gamma	E484K, S982A, N501Y *
PKamp™ VariantDetect™ SARS-CoV-2 RT-PCR Kit (Perkin Elmer)	Alpha, Beta, Gamma, Epsilon, Iota, Delta, Delta+, Kappa, Mu, Omicron	N501Y, E484K, E484Q, K417T, K417N, del69-70, L452R, P681H, P681R **
Allplex™ SARS-CoV-2 Variant I (Type H) (Seegene)	Alpha, Beta, Gamma, Eta, Iota	del69-70, E484K, N501Y *
Allplex™ SARS-CoV-2 Variant II (Type I) (Seegene)	Beta, Delta, Gamma, Kappa	W152C, L452R, K417N, K417T *
Novaplex™ SARS-CoV-2 Variants VII (Seegene)	Omicron	del69-70, E484A, N501Y *
RIDA^®^GENE SARS-CoV-2 Lineage I RUO (R-biopharm)	Alpha, Gamma, Beta, Omicron	D3L, NSP6 del106-8 *
RIDA^®^GENE SARS-CoV-2 Lineage II RUO (R-biopharm)	Delta, Kappa, Beta, Gamma, Eta, Theta	T478K, E484Q, E484K *

* depending on presence of one or more mutations.; ** five kit combination choices to detect for VOC of interest.

**Table 2:** Analytical specificity of the D950N Delta RT-PCR assay to screen for Delta VOC.

Sample Lineage	No. Samples	Confirmed by WGS	D950N Delta Assay		
			D950	D950N	Uncharacterised
VOC					
Alpha	6	6	6	ND	0
Beta	3	3	2	ND	1
Delta	28	28	ND	26	2
Omicron	32	32	32	ND	0
non-VOC					
20B, B.1.1	1	1	1	ND	0
20A, B.1	1	1	1	ND	0
B.1.466.2	3	3	3	ND	0
B.6.8	1	1	1	ND	0
Uncharacterised					
SARS-CoV-2 positive	150	n/a *	141	1	8
SARS-CoV-2 negative	61	n/a *	ND	ND	0

* Patient samples tested by Infectious Diseases Laboratory, Pathology Queensland, Queensland Health.

## Supplementary Materials

The following supporting information can be downloaded at: https://www.mdpi.com/article/10.3390/diagnostics12092056/s1, Table S1: Lowry et al. Supp Data.
